# Modulation by metformin of molecular and histopathological alterations in the lung of cigarette smoke-exposed mice

**DOI:** 10.1002/cam4.234

**Published:** 2014-03-28

**Authors:** Alberto Izzotti, Roumen Balansky, Francesco D'Agostini, Mariagrazia Longobardi, Cristina Cartiglia, Rosanna T Micale, Sebastiano La Maestra, Anna Camoirano, Gancho Ganchev, Marietta Iltcheva, Vernon E Steele, Silvio De Flora

**Affiliations:** 1 Department of Health Sciences, University of GenoaGenoa, Italy; 2 National Center of OncologySofia, 1756, Bulgaria; 3 National Cancer InstituteRockville, Maryland

**Keywords:** Cigarette smoke, DNA damage, lung tumors, metformin, microRNAs

## Abstract

The anti-diabetic drug metformin is endowed with anti-cancer properties. Epidemiological and experimental studies, however, did not provide univocal results regarding its role in pulmonary carcinogenesis. We used Swiss H mice of both genders in order to detect early molecular alterations and tumors induced by mainstream cigarette smoke. Based on a subchronic toxicity study, oral metformin was used at a dose of 800 mg/kg diet, which is 3.2 times higher than the therapeutic dose in humans. Exposure of mice to smoke for 4 months, starting at birth, induced a systemic clastogenic damage, formation of DNA adducts, oxidative DNA damage, and extensive downregulation of microRNAs in lung after 10 weeks. Preneoplastic lesions were detectable after 7.5 months in both lung and urinary tract along with lung tumors, both benign and malignant. Modulation by metformin of 42 of 1281 pulmonary microRNAs in smoke-free mice highlighted a variety of mechanisms, including modulation of AMPK, stress response, inflammation, NF*κ*B, Tlr9, Tgf, p53, cell cycle, apoptosis, antioxidant pathways, Ras, Myc, Dicer, angiogenesis, stem cell recruitment, and angiogenesis. In smoke-exposed mice, metformin considerably decreased DNA adduct levels and oxidative DNA damage, and normalized the expression of several microRNAs. It did not prevent smoke-induced lung tumors but inhibited preneoplastic lesions in both lung and kidney. In conclusion, metformin was able to protect the mouse lung from smoke-induced DNA and microRNA alterations and to inhibit preneoplastic lesions in lung and kidney but failed to prevent lung adenomas and malignant tumors induced by this complex mixture.

## Introduction

The biguanide metformin became available as a glucose-lowering drug in the late 1950s. It was approved by the U.S. FDA in 1995, and is at present the most widely prescribed drug in the world for the treatment of type 2 diabetes 1–3. Metformin represses hepatic gluconeogenesis, lowers circulating insulin and decreases insulin resistance by activating AMP-activated protein kinase (AMPK). This is the primary downstream kinase regulated by the tumor suppressor gene *Liver Kinase B1* (*LKB1*), which results in an inhibition of the mammalian target of rapamycin (mTOR) 4. Several other mechanisms have been proposed as well 2.

In recent years, growing attention has been paid to the possibility that metformin may also act as an anti-cancer drug and may thus be proposed as a potential cancer chemopreventive agent. Its protective role depends on the fact that, for a variety of reasons, type 2 diabetes has been associated with certain cancers 5–7. In addition, due to the pleiotropic properties of this drug, metformin interferes with various mechanisms involved in the carcinogenesis process (see Discussion).

The results of epidemiological studies evaluating the cancer protective properties of metformin are rather controversial, and the results of meta-analyses in diabetic patients treated with this drug are not univocal. For instance, Decensi et al. 8 selected 11 studies and reported a 31% reduction in the overall relative risk in subject taking metformin compared to other anti-diabetic drugs, with a significant inverse association limited to pancreatic cancer and hepatocellular cancer. Likewise, in 11 studies selected by Noto et al. 9, a significant decrease in both incidence and mortality for all cancers was reported among metformin users, with significant decreases of incidence for colorectal cancer, hepatocellular cancer, and lung cancer. In contrast, Stevens et al. 10 did not find any significant effect of metformin on all-cancer mortality in the meta-analysis of 14 randomized controlled trials. Further on, from the analysis of 37 studies, Zhang et al. 11 concluded that there is a weak but statistically significant decrease in overall cancer incidence and mortality among metformin users, with significant decreases in both incidence and mortality regarding liver cancer and breast cancer and in incidence only regarding pancreatic cancer and colorectal cancer. In addition, a wealth of experimental studies have shown that metformin is able to inhibit cell growth in cancer cell lines from a variety of tissues as well as in xenografts of human cancers implanted in nude mice 1.

Focussing on the relationships between metformin and pulmonary carcinogenesis, an observational cohort study in 4085 metformin users showed a decreased incidence of lung cancer, which however was no longer detectable by adjusting the data for various confounding factors, among which are smoking habits 12. Similarly, a case–control study led to the conclusion that metformin does not alter the risk of lung cancer in diabetic patients 13. In A/J mice treated parenterally with the tobacco-specific nitrosamine 4-(methylnitrosamino)-1-(3-pyridyl)-1-butanone (NNK), metformin was able to reduce the multiplicity of surface lung tumors when given both orally and intraperitoneally 14. In 129/Sv mice treated parenterally with urethane, oral metformin slightly but significantly inhibited the incidence of total tumors (lung adenomas and thymic lymphomas) but did not significantly affect the incidence of solid or trabecular lung adenomas 15.

Inducing lung tumors in laboratory animals exposed to cigarette smoke (CS), as a complex mixture, is a difficult task. We demonstrated that mainstream CS (MCS) becomes convincingly carcinogenic in Swiss H mice when exposure starts at birth 16, which compares favorably to exposure during adulthood 17. We showed that this animal model is suitable to evaluate the efficacy and safety of chemopreventive agents under conditions mimicking (1) an intervention in current smokers, as evaluated by testing the following drugs or natural compounds: phenethyl isothiocyanate (PEITC), budesonide and *N*-acetylcysteine (NAC) 18, berry extracts 19, myo-inositol, vorinostat, the anti-diabetic drug pioglitazone, bexarotene, and a combination of pioglitazone and bexarotene 20, lapatinib, licofelone and celecoxib (manuscript in preparation); (2) an intervention in ex-smokers, as evaluated by testing PEITC and budesonide 18, lapatinib and celecoxib (manuscript in preparation); (3) a transplacental intervention, as evaluated by administering the antioxidants NAC and ascorbic acid throughout pregnancy 21,22. Furthermore, using the same mouse strain, we investigated the interplay between exposure to MCS, histopathological alterations, and treatment with cancer chemopreventive agents in defining microRNA (miRNA) profiles in lung 23 and evaluated the relationship between pulmonary miRNA expression and proteome profiles, systemic cytogenetic damage and lung tumors as related to exposure to MCS and treatment with chemopreventive agents 20. In this study, we applied this murine model in order to explore the molecular and histopathological alterations induced by MCS and their modulation by oral metformin. Parallel studies were carried out in two laboratories, using the same mouse strain and method of exposure to MCS. The laboratory in Genoa performed the subchronic toxicity study and the study on evaluation of molecular biomarkers, including bulky DNA adducts, oxidative DNA damage, and miRNA profiles in both smoke-free and MCS-exposed mice. The laboratory in Sofia evaluated modulation by metformin of systemic genotoxic damage, lung tumors, and other histopathological alterations.

The results obtained provide evidence that metformin regulates the expression of a number of miRNAs in the lung of smoke-free mice, highlighting its multiple mechanisms of action, and exerts some protective effects toward molecular and histopathological alterations induced by MCS. The drug prevented MCS-induced preneoplastic lesions in both lung and kidney but did not affect the yield of lung tumors.

## Materials and Methods

### Mice

Strain H mice, originated from Swiss albino mice, were obtained from the Animal Laboratory of the National Center of Oncology (Sofia, Bulgaria). Newborn mice of this strain are sensitive to the induction of lung tumors by MCS 16–18,20–23. One hundred postweanling mice (50 males and 50 females) were used in the Genoa laboratory for the subchronic toxicity study. In the same laboratory, 40 newborn mice (20 males and 20 females) were used for evaluating the effect of metformin on intermediate molecular biomarkers in the lung. A total of 277 newborn mice (137 males and 140 females) were used in the Sofia laboratory for evaluating lung tumors and other histopathological lesions, and a subgroup of 60 mice was used for evaluating the systemic genotoxicity.

The mice were housed in Makrolon™ cages on sawdust bedding and maintained on standard rodent chow (Teklad 2018; Harlan Laboratories in Genoa and Kostinbrod in Sofia) and drinking water ad libitum. The animal room temperature was 23 ± 2°C and the relative humidity was 55%, with a 12 h day–night cycle. Housing, breeding, and treatment of mice were in accordance with NIH and European (86/609/EEC Directive) guidelines.

### Chemopreventive agent

Metformin (*N*,*N*-dimethylimidodicarbonimidic diamide) was supplied by NCI via MRIGlobal (Kansas City, MO).

### Exposure to MCS

A whole-body exposure of mice to MCS was achieved by burning 3R4F Kentucky reference cigarettes (University of Kentucky, Lexington, KY) in the Genoa laboratory and commercially available cigarettes (Melnik King Size; Bulgartabac) in the Sofia laboratory, as previously described 16–22. MCS was generated by drawing 15 consecutive puffs, each of 60 mL and lasting 6 sec, by using a syringe connected with the exposure chamber. Each daily session of treatment with MCS involved six consecutive exposures, lasting 10 min each, with 1-min intervals during which a total air change was made. 3R4F cigarettes have a declared content of 9.4 mg tar and 0.7 mg nicotine and deliver 12 mg CO each, whereas Melnik cigarettes have a declared content of 9 mg tar and 0.8 mg nicotine and deliver 10 mg CO each. The average concentrations of total particulate matter in the exposure chambers were 684 and 547 mg/m^3^, respectively.

### Subchronic toxicity study

Metformin was incorporated in the diet at four dose levels (125, 250, 500, and 1000 mg/kg diet). These doses were selected based on literature data in mouse studies and taking into account the therapeutical doses used in humans. Each metformin dose and the corresponding metformin-free control were administered to 20 postweanling mice (10 males and 10 females). The mice were inspected daily for general appearance and behavior and were weighed at weekly intervals for 6 weeks. The MTD (maximum-tolerated dose) was assumed as the highest dose of the drug that did not produce visible alterations and did not alter the body weight by more than 10%.

### Evaluation of molecular intermediate biomarkers in the lung

Four groups of newborn mice, each composed of 10 mice (five males and five females), were used for evaluating molecular biomarkers, including bulky DNA adducts, oxidative DNA damage, and miRNA expression profiles in the lung, as follows. *Group A*: mice kept in filtered air for 10 weeks (sham-exposed mice); *Group B*: mice exposed to MCS for 10 weeks, starting within 12 h after birth (MCS-exposed mice); *Group C*: mice receiving metformin (800 mg/kg diet) for 6 weeks, starting after weaning (4 weeks); *Group D*: MCS-exposed mice receiving metformin after weaning until the end of the experiment.

The mice were inspected daily and weighed at weekly intervals. At 10 weeks of age all mice were sacrificed by CO_2_ asphyxiation and their lungs were collected. The left lung, to be used for DNA analyses, was frozen at −80°C. The right lung, to be used for miRNA analyses, was immersed in RNA*later*® solution for 24 h and then transferred at −80°C.

DNA was extracted individually from the lung of all 40 mice and purified by using a commercially available kit (GenElute™ Mammalian Genomic DNA Miniprep kit; Sigma, St. Louis, MO). Spectrophotometric analyses showed that the DNA quality was satisfactory, the 260/280 ratio being 1.9 ± 0.04 (mean ± SD). Bulky DNA adducts were measured by ^32^P postlabeling as previously described 24. A blank and a positive control (BPDE-dG) were also tested. The same lung DNA preparations were assayed for 8-hydroxy-2′-deoxyguanosine (8-oxo-dGuo) as previously described 24.

For RNA extraction, the 10 lung specimens collected from sham-exposed mice and those collected from MCS-exposed mice were processed individually in order to evaluate the interindividual variability, whereas the specimens from the Metformin and MCS + Metformin groups were processed as two pools from five mice each, one for males and one for females. RNA was extracted by using Triazol and column chromatography. Quantification of RNA and evaluation of its integrity were performed as previously described 25.

MiRNA analyses were performed by using the seventh-generation miRCURY LNA™ microRNA Array (Exiqon, Woburn, MA), which contains 3100 capture probes covering human, mouse and rat miRNAs. In particular, this array covers 1281 mouse miRNAs, which represent the 88.6% of the mouse miRNAs listed in miRBase 19. The miRNA microarray data are available at GEO database (http://www.ncbi.nlm.nih.gov/GEO/, GEO number requested).

In addition, the expression of two miRNAs (*let-7f* and *miR-30b*) was validated by real-time quantitative polymerase chain reaction (qPCR), as previously described 26. The specificity of the qPCR amplified products was confirmed by analyzing melting temperature peaks, which were 70.5°C for *let-7f* and 72.0°C for *miR-30b*.

### Evaluation of systemic cytogenetic damage, lung tumors, and other histopathological alterations

This study included three groups of newborn mice, as follows. *Group A*: 94 mice (45 males and 49 females) kept in filtered air for 7.5 months (sham-exposed mice); *Group B*: 109 mice (55 males and 54 females) exposed to MCS for 4 months, starting within 12 h after birth, and then kept in filtered air for an additional 3.5 months (MCS-exposed mice); *Group C*: 74 MCS-exposed mice (37 males and 37 females) receiving metformin (800 mg/kg diet), starting after weaning until the end of the experiment.

At 4 months, immediately after discontinuing exposure to MCS, peripheral blood was collected from the tail lateral vein from 20 mice (10 males and 10 females) per each one of the three experimental groups and smeared onto slides (two slides/mouse). After staining with May-Grünwald-Giemsa, the frequency of micronucleated (MN) normochromatic erythrocytes (NCE) was scored by analyzing microscopically 50,000 NCE/mouse.

The mice that became sick or moribund before the end of the experiment were separated and isolated from the other mice of the same cage. All mice surviving at 7.5 months of age were killed by CO_2_ asphyxiation. A complete necropsy of both mice that died prematurely and mice sacrificed after 7.5 months was performed. Lungs, liver, kidney, urinary bladder, and all organs with suspected macroscopical lesions were fixed in 10% formalin, cut in standardized sections and stained with hematoxylin and eosin. In particular, the accessory, middle and caudal lobes of the right lung were cut into two pieces each, whereas the cranial lobe was left uncut. The left lung was cut into three pieces. This accounted for a total of 10 lungs sections to be subjected to standard histopathological analyses.

### Data processing and statistical analysis

After local background subtraction, microarray data were log transformed, normalized, and analyzed by GeneSpring® software version 7.2 (Silicon Genetics, Redwood City, CA). Expression data were median centered by using the GeneSpring normalization option. Comparisons between experimental groups were done by evaluating the fold variations of quadruplicate data generated for each miRNA. In addition, the statistical significance of the differences was evaluated by means of the GeneSpring ANOVA applied by using Bonferroni multiple testing corrections. As inferred from volcano-plot analysis, differences with *P* < 0.05 and >2.0-fold variations between experimental groups were taken as significant.

Comparisons between groups regarding survival of mice and incidence of histopathological lesions were made by *χ*^2^ analysis. Body weights, frequency of MN NCE, and multiplicity of preneoplastic and neoplastic lesions were expressed as means ± SE of the mice composing each experimental group, and comparisons between groups were made by Student's *t*-test for unpaired data.

## Results

### Subchronic toxicity study

**Figure 1:**
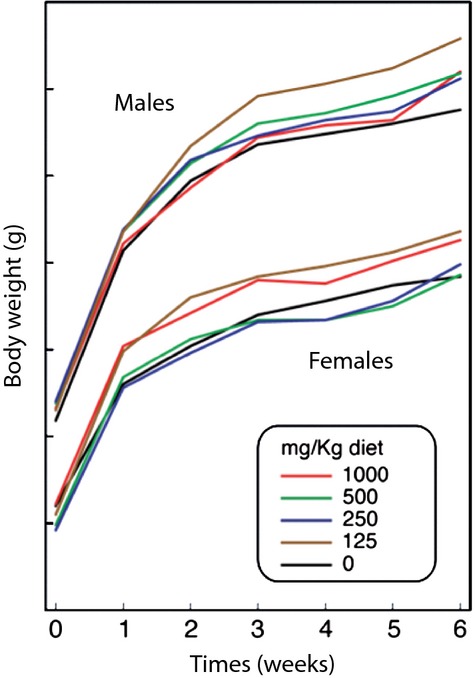
Body weight gain of Swiss H male and female mice receiving varying doses of metformin with the diet for 6 weeks.

All 100 postweanling mice and their controls (50 males and 50 females) survived during the 6 weeks of treatment with metformin, given with the diet at four dose levels. The daily inspection did not reveal any sufferance or behavioral alterations. Figure 1 shows the body weights, measured at weekly intervals in male and female mice treated with metformin, as compared with untreated controls. At any time and dose, metformin did not affect the body weight gain observed in controls by more than 10%. Hence, in all subsequent experiments we used metformin at 800 mg/kg diet, which is the 80% of the highest tested dose.

### Body weights of the mice used for evaluating molecular biomarkers

Exposure of mice to MCS for 10 weeks, starting at birth, resulted in a significant decrease in body weights from the seventh week of life onwards in males and from the eighth week of life onwards in females. Administration of metformin to MCS-exposed mice, starting after weaning (∼4 weeks) had moderate protective effect in females, in the sense that the body weights were not significantly different from sham-exposed mice any longer, although the difference with MCS-exposed mice was not statistically significant (see Table S1).

### Bulky DNA adducts in lung

**Figure 2:**
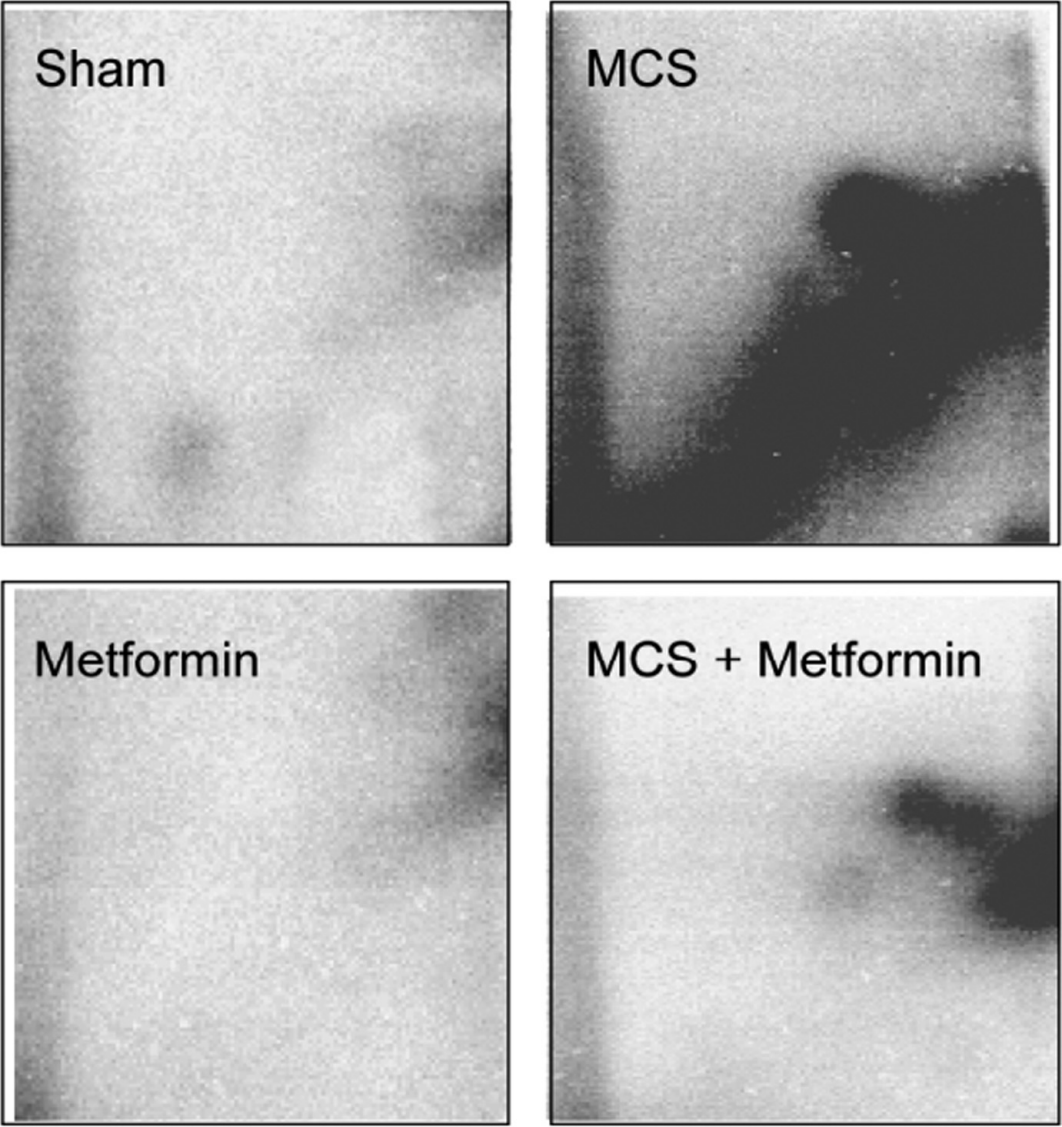
Examples of ^32^P autoradiographs obtained by testing the lung DNA of mice as related to exposure to MCS and/or treatment with metformin. MCS, mainstream cigarette smoke.

As shown in the examples reported in Figure 2, exposure of mice to MCS resulted in the formation of a typical diagonal radioactive zone in ^32^P autoradiographs, which was considerably attenuated by metformin administration. Table 1 reports DNA adduct levels in mouse lung as related to exposure to MCS and treatment with metformin. MCS considerably increased DNA adduct levels as compared with Sham in both genders (13.8-fold in males and 16.7-fold in females). Metformin had no effect on “spontaneous” DNA adducts and significantly decreased MCS-induced adducts (2.6-fold in males and 3.4-fold in females).

**Table 1:** Bulky DNA adducts and 8-oxo-dGuo evaluated by ^32^P postlabeling in mouse lung

Treatment	Gender	Adducts/10^8^ nucleotides	8-oxo-dGuo/10^5^ nucleotides
Sham	M	1.3 ± 0.20	1.7 ± 0.25
	F	1.2 ± 0.17	2.1 ± 0.20
	M + F	1.3 ± 0.12	1.9 ± 0.19
Metformin	M	1.3 ± 0.20	2.3 ± 0.13
	F	1.0 ± 0.15	2.5 ± 0.30
	M + F	1.2 ± 0.13	2.4 ± 0.09
MCS	M	17.9 ± 1.77^b^	5.0 ± 0.08^b^
	F	20.0 ± 3.30^b^	6.0 ± 0.44^b^
	M + F	18.9 ± 1.59^b^	5.5 ± 0.26^b^
MCS + Metformin	M	6.9 ± 1.58^a^,^c^	3.6 ± 0.27^b^,^c^
	F	5.8 ± 1.04^a^,^c^	3.9 ± 0.31^b^,^c^
	M + F	6.3 ± 0.92^a^,^d^	3.7 ± 0.20^b^,^d^

The data are means ± SE of the results obtained in five mice/treatment/gender. MCS, mainstream cigarette smoke.

a *P* < 0.01 and

b *P* < 0.001, as compared with sham-exposed mice of the same gender;

c *P* < 0.01 and

d *P* < 0.001, as compared with MCS-exposed mice.

### Oxidative DNA damage in lung

As shown in Table 1, exposure of mice to MCS resulted in significant increases in 8-oxo-dGuo levels in the lung of both males and females as compared with sham-exposed mice (2.9-fold in both genders). Administration of metformin to sham-exposed mice did not affect oxidative DNA damage, whereas its administration to MCS-exposed mice significantly decreased 8-oxo-dGuo levels in both males (1.4-fold) and females (1.5-fold).

### Expression of pulmonary miRNAs by microarray

**Figure 3:**
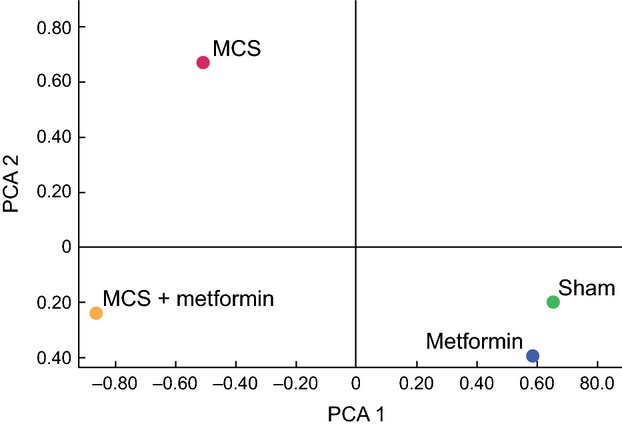
Principal component analysis showing the overall expression of 1281 pulmonary miRNAs in sham-exposed mice, mice receiving metformin with the diet, MCS-exposed mice, and MCS-exposed mice treated with metformin. MCS, mainstream cigarette smoke.

As compared with sham-exposed mice, metformin dysregulated 42 of 1281 pulmonary miRNAs (3.7%), 6 of which were downregulated and 36 were upregulated by this drug. However, the modulated miRNAs were mainly expressed at low levels of intensity. Bidimensional principal component analyses (Fig. 3) confirmed that no dramatic difference occurred in the overall miRNA profiles of sham-exposed and metformin-treated mice, which fell in the same quadrant. In particular, Table 2 (left column) provides a list of the miRNAs that were modulated by metformin in sham-exposed mice. The table indicates the direction of regulation and its intensity, as inferred from the Metformin/Sham ratio. The picture of the main functions of the metformin-dysregulated miRNAs is quite complex and embraces a large variety of mechanisms involved in the carcinogenesis process, sometimes with contrasting regulations of the same mechanisms by different miRNAs, which is likely to reflect a fine tuning regulation. The most frequently targeted functions were *Ras*, *Myc*, *Tgf-β*, NF*κ*B, stress response, vesicle trafficking, cell proliferation, apoptosis, and inflammation.

**Table 2:** Identification and main functions of miRNAs that were either dysregulated by metformin in sham-exposed mice or restored by metformin in MCS-exposed mice

	Fold-variation		
		
miRNA	Metformin versus Sham	Metformin + MCS versus MCS	Function
*let-7f*	↑3.44	↑2.65	Cell proliferation, *Ras* activation, angiogenesis
*miR-1a*	↑2.70		NA
*miR-17*	↑2.14	↑3.57	Oncogene (*Pten*, *Dicer*, *Tgf-β*, *Myc*) suppressor
*miR-26a*	↑2.67	↑4.39	*Tgf-β* suppressor
*miR-26b*	↑2.51	↑2.32	*Tgf-β* suppressor
*miR-30b*	↑3.83	↑2.03	AMPK modulation, intercellular adhesion, protein repair, NF*κ*B activation, cell cycle, EGF activation, stem cell recruitment, multidrug resistance
*miR-98*	↑3.34		Angiogenesis, apoptosis, oncogene (*Fus*) suppression
*miR-126*	↓2.07		Gene transcription
*miR-129*	↑3.23		Calmodulin transcription activation
*miR-135b*	↑2.66	↑3.54	*Ras* regulation, cell adhesion
*miR-138*	↑2.40		Oncogene *Myc* activation, inflammation, *p53* suppression, inhibition of retinoic acid receptor
*miR-143*	↑2.69		Oncogene (*Apc*, *Ras*) suppression, cell differentiation
*miR-148b*	↑2.14		AMPK modulation
*miR-193*	↓2.06		Signal transduction
*miR-299a*	↓2.40		NF*κ*B activation, stress response, peroxisome activation
*miR-342*	↑3.28		Stress response, protein repair
*miR-350*	↑2.48		Muscle hypertrophy, apoptosis
*miR-362*	↑2.94		Cell cycle arrest
*miR-376c*	↑2.55		Carbonic anhydrase (antioxidant), peroxisome biogenesis, *p53*, cell cycle progression, intracellular vesicle trafficking
*miR-382*	↑4.14	↑2.07	Gene transcription
*miR-466 h*	↑3.09		Cell proliferation, *Ras* activation, gene transcription
*miR-470*	↑2.28		Oncogene (*Ras*) suppression, intracellular vesicle trafficking, xenobiotic metabolism
*miR-487b*	↑2.08	↑3.15	Oncogene (*Myc, Ras*) suppression
*miR-490*	↓2.01		Stress response, cell proliferation
*miR-542*	↓2.60		NA
*miR-574*	↑2.09		Inflammation (Tlr9 activation), cell proliferation, apoptosis
*miR-669j*	↑3.12		NA
*miR-672*	↑2.40		NA
*miR-674*	↑2.19		NA
*miR-744*	↑4.35		Oncogene (*Tgf*) suppression
*miR-873*	↑2.53	↑3.22	NA
*miR-1930*	↑3.31		NA
*miR-1934*	↑2.17	↑3.27	NA
*miR-1942*	↑2.49		NA
*miR-3064*	↑2.50		NA
*miR-3065*	↑3.20		NA
*miR-3069*	↑2.98		NA
*miR-3071*	↑3.51		NA
*miR-3073*	↓2.78		NA
*miR-3092*	↑3.48		NA
*miR-3093*	↑3.28		NA
*miR-3109*	↓2.07		NA

All reported variations were statistically significant (*P* < 0.05). Upward and downward arrows indicate upregulation and downregulation, respectively. MCS, mainstream cigarette smoke; NA, not available.

MCS mainly affected miRNA expression in the sense of downregulation, and MCS fell in an opposite quadrant at principal component analysis, as compared with both Sham and Metformin. In particular, 62 (4.8%) of the 1281 miRNAs tested were significantly downregulated in the lung of MCS-exposed mice.

Metformin was effective in changing the miRNA alterations resulting from exposure to MCS, as confirmed by principal component analysis (Fig. 3). In fact, MCS + Metformin was allocated in a quadrant different from both Sham and MCS. Table 2 (right column) shows the list of the 10 MCS-downregulated miRNAs whose expression was normalized by metformin treatment. It is noteworthy that all these miRNAs had been upregulated by metformin also in sham-exposed mice. Their main functions pertain to the cell cycle regulation, intercellular adhesion, protein repair, angiogenesis, stem cell recruitment, multidrug resistance, and modulation of *Ras*, *Myc*, *Pten*, *Tgf-β*, NF*κ*B, and EGF.

### Validation of *let-7f* and *miR-30b* microarray results by real-time qPCR

Because of the biological relevance of two metformin-modulated miRNAs, *let-7f* and *miR-30b*, their expression was validated by qPCR analysis. qPCR amplification products were quantified by taking into account the number of the first positive amplification cycle and by making reference to the amount of 5S rRNA used as an internal reference standard. For *let-7f*, the normalized fluorescent intensity was 45.3 Fluorescence Units (FU) in the lung of MCS-exposed mice, in the absence of metformin, and 94.6 FU in the lung of MCS-exposed mice treated with metformin. Accordingly, qPCR analysis demonstrated that metformin upregulated 2.1-fold *let-7f* in MCS-exposed mice, which is in line with microarray results indicating a 2.7-fold *let-7f* upregulation in the same experimental groups (Table 2). For *miR-30b*, the normalized fluorescent intensity was 20.5 FU in the lung of sham-exposed mice, in the absence of metformin, and 61.3 FU in the lung of mice treated with metformin. Accordingly, qPCR analysis demonstrated that metformin upregulated 3.0-fold *let-7f*, which is in line with microarray results indicating a 3.8-fold *miR-30b* upregulation in the same experimental groups (Table 2).

### Survival and body weights of the mice used for evaluating clastogenicity and histopathological alterations

A total of 277 mice (137 males and 140 females) were available for this part of the study. Table S2 shows survival and body weights of the mice belonging to the three experimental groups (Sham, MCS, and MCS + metformin) at 1 month of life, when metformin started to be administered, at 4 months, when exposure to MCS was discontinued, and at 7 months, 15 days before sacrifice of all surviving mice. MCS did not affect survival of mice at any time but caused a significant decrease in body weights at 1 and 4 months of life, during the period of exposure. The body weight gain was recovered 3 months later. Administration of metformin to MCS-exposed mice did not affect the MCS-related variations of body weight, excepting a slight but significant decrease at 7 months in males.

### Clastogenicity in peripheral blood erythrocytes

A total of 3 million NCE (50,000 NCE in 60 mice) were scored at the microscope at 4 months of life. The results of these analyses are summarized in Table S2. Irrespective of administration of metformin, exposure of mice to MCS resulted in a significant increase in MN NCE in both males and females.

### Lung tumors and other histopathological alterations

The results of histopathological analyses relative to all 277 mice are summarized in Table 3. Exposure of mice to MCS for 4 months, starting at birth, followed by 3.5 months in filtered air, resulted in a number of significant changes in the lung. We refer to previous papers 16,17, for examples showing the morphological appearance of these lesions. The observed changes included increases in the incidences of emphysema, alveolar epithelial hyperplasia, blood vessel proliferation, microadenomas, adenomas, and malignant tumors. As shown in Table 3, the multiplicity of preneoplastic and neoplastic lesions was also significantly increased in MCS-exposed mice of both genders. Of the 36 foci of malignant tumors detected in the lung of MCS-exposed mice, which were totally absent in sham-exposed mice, the majority were adenosquamous carcinomas (72.2%), followed by squamocellular carcinomas and bronchoalveolar carcinomas (8.3% each), and by carcinomas in situ and adenocarcinomas (5.6% each). In the urinary tract, MCS caused a significant increase in the incidence of tubular epithelial hyperplasia in the kidney of both males and females as well as a selective induction of papillary epithelial hyperplasia in the urinary bladder of males.

**Table 3:** Incidence (%) and multiplicity (mean ± SE) of histopathological alterations in organs of Swiss H mice, as related to exposure to MCS and treatment with metformin

Organ Histopathological alteration	Gender	Sham (45M + 49F)	MCS (55M + 54F)	MCS + metformin (37M + 37F)
Lung				
Emphysema	M	1 (2.2%)	6 (10.9%)	2 (5.4%)
Incidence	F	0	9 (16.7%)^b^	2 (5.4%)
	M + F	1 (1.1%)	15 (15.0%)^c^	4 (5.4%)
Alveolar epithelial hyperplasia	M	2 (4.4%)	17 (30.9%)^c^	12 (32.4%)^c^
Incidence	F	2 (4.1%)	14 (25.9%)^b^	12 (32.4%)^c^
	M + F	4 (4.3%)	31 (28.4%)^c^	24 (32.4%)^c^
Bronchial epithelial hyperplasia	M	1 (2.2%)	4 (7.3%)	4 (10.8%)
Incidence	F	0	3 (5.6%)	1 (2.7%)
	M + F	1 (1.1%)	7 (6.4%)	5 (6.8%)
Blood vessel proliferation and hemangiomas	M	2 (4.4%)	5 (9.1%)	6 (16.2%)
Incidence	F	1 (2.0%)	6 (11.1%)^a^	1 (2.7%)
	M + F	3 (3.2%)	13 (11.9%)^a^	7 (9.5%)
Microadenomas	M	0	32 (58.2%)^c^	9 (24.3%)^c^,^d^
Incidence	F	0	29 (52.7%)^c^	17 (45.9%)^c^
	M + F	0	61 (55.9%)^c^	26 (35.1%)^c^,^d^
Multiplicity (mean ± SE)	M	0	7.7 ± 1.32^c^	1.9 ± 0.60^c^,^e^
	F	0	10.2 ± 1.57^c^	7.2 ± 1.72^c^
	M + F	0	8.9 ± 1.03^c^	4.5 ± 0.95^c^,^d^
Adenomas	M	1 (2.2%)	14 (25.5%)^b^	13 (35.1%)^c^
Incidence	F	2 (4.1%)	13 (24.1%)^b^	10 (27.0%)^c^
	M + F	3 (3.2%)	27 (24.8%)^c^	23 (31.1%)^c^
Multiplicity (mean ± SE)	M	0.04 ± 0.04	4.1 ± 1.13^c^	7.2 ± 1.95^c^
	F	0.06 ± 0.05	2.5 ± 1.03^c^	4.7 ± 0.58^c^
	M + F	0.05 ± 0.03	3.3 ± 0.76^c^	5.9 ± 1.25^c^
Malignant tumors	M	0	7 (12.7%)^a^	5 (13.5%)
Incidence	F	0	6 (11.1%)^a^	4 (10.8%)
	M + F	0	13 (11.9%)^c^	9 (12.2%)
Multiplicity (mean ± SE)	M	0	0.4 ± 0.16^a^	0.4 ± 0.21^a^
	F	0	0.3 ± 0.15^a^	0.4 ± 0.21^a^
	M + F	0	0.3 ± 0.11^b^	0.4 ± 0.15^b^
Liver				
Parenchymal degeneration	M	1 (2.2%)	3 (5.5%)	1 (2.7%)
Incidence	F	1 (2.0%)	3 (5.6%)	0
	M + F	2 (2.1%)	6 (5.5%)	1 (1.4%)
Kidney				
Tubular epithelial hyperplasia	M	1 (2.2%)	16 (29.1%)^c^	0^c^
Incidence	F	0	13 (24.1%)^c^	4 (10.8%)^a^
	M + F	1 (1.1%)	29 (26.6%)^c^	4 (5.4%)^e^
Urinary bladder				
Papillary epithelial hyperplasia	M	1 (2.2%)	7 (12.7%)	4 (10.8%)
Incidence	F	0	0	0
	M + F	1 (1.1%)	7 (6.4%)^a^	4 (5.4%)

MCS, mainstream cigarette smoke.

Statistical analysis:

a *P* < 0.05,

b *P* < 0.01, and

c *P* < 0.001, as compared with Sham;

d *P* < 0.01 and

e *P* < 0.001, as compared with MCS-exposed mice of the same gender, in the absence of metformin.

Administration of metformin to MCS-exposed mice did not significantly affect the yield of MCS-induced lung adenomas and malignant tumors. However, it caused a significant decrease in both incidence and multiplicity of microadenomas, especially in males. In addition, the MCS-induced tubular epithelial hyperplasia in kidney was totally suppressed by metformin in males.

## Discussion

The results of this study demonstrate that oral metformin is able to modulate the expression of a number of miRNAs in the lung of smoke-free mice and to counteract some adverse effects produced by MCS in both lung and extrapulmonary tissues. The dose of metformin used in our studies was not far away from the dose used in humans. In fact, the initial therapeutical dosage of metformin in diabetic patients is 1000 mg/day, which may be increased up to 2500 mg/day, corresponding to 1.25 mg in a 35 g mouse. Assuming that each mouse eats 5 g diet/day, this figure would account for 250 mg metformin/kg diet, which was higher or equal to the two lowest doses assayed in our subchronic toxicity study. Since the maximum tested dose (1000 mg/kg diet) did not produce any macroscopically evident adverse effect, we precautionally used in all studies the dose of 800 mg/kg diet, which is just 3.2 times higher than the therapeutical dose in humans.

In agreement with the conclusions of our previous studies in neonatal Swiss H mice exposed to MCS during the first 4 months of life 16–18,20–22, MCS caused the formation of histopathological alterations in the lung, also including preneoplastic and neoplastic lesions, both benign and malignant. In addition, MCS damaged the urinary tract by significantly increasing the incidences of both tubular epithelial hyperplasia in the kidney and papillary epithelial hyperplasias in the urinary bladder. Interestingly, the latter lesion was selectively induced in males and not in females, which confirms the results of a previous study 27 and is consistent with the recognized influence of sex hormones in modulating development of bladder cancer 28. Keeping in mind the prominent role of chronic inflammation in CS-related carcinogenesis 29,30, an important mechanism explaining the lower susceptibility of females is that estrogens are able to reduce chronic bladder inflammation 31.

Besides histopathological alterations, MCS induced a systemic clastogenic effect and molecular alterations in the lung. The choice of the investigated molecular end-points was based on our previous experience in studies using rodents exposed to CS 20,23–25,31–33. In particular, MCS caused an intense formation of DNA adducts and induction of oxidative DNA damage. Furthermore, MCS extensively downregulated lung miRNAs, which reflects both adaptive mechanisms and activation of a variety of pathways involved in pulmonary carcinogenesis. These molecular effects have previously been discussed 20,23,25,31–33. Clearly, since DNA analyses and RNA analyses require a specific preparation of samples, these end-points had to be evaluated in different lungs from each mice.

So far, modulation of miRNA expression by metformin had been investigated in vitro in prostatic cancer cell 34, pancreatic cancer cells 35,36, and breast cancer cells 37. We analyzed the large majority of the mouse miRNAs identified so far in the lung of mice receiving oral metformin from weaning to the tenth week of life. The drug modulated a number of pulmonary miRNAs but the effects were not dramatic, the overall profiles in metformin-treated mice being close to those recorded in sham-exposed mice. The resulting data contribute to clarify a variety of mechanisms of metformin under in vivo conditions.

In line with the known role of this drug to activate AMPK 4, considered an ideal drug target for cancer treatment 38, metformin upregulated both *miR-148b*, which targets this kinase, and *miR-30b*, belonging to a family of miRNAs that are known to modulate AMPK 39. AMPK is a known inhibitor of TGF 40, which was targeted by three miRNAs (*miR-26a*, *miR-26b*, and *miR-744*) that were upregulated by metformin in mouse lung. Metformin also upregulated *let-7f*, whose expression is boosted by Lin28 and/or Lin28b activities, known to be antagonized by this drug 41. In addition, metformin modulated the expression of a number of miRNAs (*let-7f*, *miR-30b*, *miR-362*, *miR-376c*, *miR-466h*, *miR-490*, and *miR-574*) involved in the regulation of the cell cycle, which is a crucial mechanism in the AMPK-mediated activity of this drug 42. Modulation of pulmonary miRNAs targeting *p53* (*miR-138* and *miR-376c*) and apoptosis (*miR-98* and *miR-350*) is consistent with the notion that AMPK is involved in the p53-mediated cell cycle arrest and apoptosis 2.

Several miRNAs upregulated in the lung of metformin-treated mice, including *miR-30b*, *miR-138*, *miR-239a*, *miR-342*, and *miR-574*, are involved in stress response and inflammation and target NF*κ*B or Tlr9 (Toll-like receptor). Tlr9 activity is mediated by mTOR signaling 43, which is an important and early mechanism involved in CS-related carcinogenesis 14. It is also established that metformin inhibits the formation of reactive oxygen species (ROS) by oncogenic *Ras* 42. In our in vivo study, the expression of miRNAs targeting *Ras* (*let-7f*, *miR-135b*, *miR-143*, *miR-466h*, *miR-470*, and *miR-487b*) was regulated in the lung of metformin-treated mice, along with another miRNA (*miR-376c*) playing an antioxidant role. Consistently with the finding that metformin reduced endogenous ROS and the associated DNA damage 44, we found that the oral administration of this anti-diabetic drug results in a sharp inhibition of 8-oxo-dGuo and bulky DNA adduct levels in the lung of MCS-exposed mice.

The study of miRNA expression in breast cancer cell lines led to the conclusion that *Dicer* modulation and c-*Myc* targeting play an important role in the anti-cancer metabolic effects of metformin 37. We confirmed these findings in vivo, since metformin upregulated *miR-17* in mouse lung. Furthermore, this drug modulated miRNAs that target angiogenesis (*let-7f* and *miR-98*), stem cell recruitment, and multidrug resistance (*miR-30b*). These data are consistent with the findings that metformin inhibited endothelial cell migration and angiogenesis, an effect that was partially AMPK dependent 45, reduced the sizes and number of mammospheres and expression of the estrogen receptor-mediated OCT4 in human breast carcinoma stem cells 46, and inhibited the anti-inflammatory response associated with cellular transformation and cancer stem cell growth 47. Accordingly, this anti-diabetic drug has been proposed as an emerging option for targeting cancer stem cells and metastasis 1. Indeed, metformin was found to prevent the development of oral squamous cell carcinomas from carcinogen-induced premalignant lesions in mice 48. A proportion of the miRNAs that were upregulated by metformin in the lung of smoke-free mice were also upregulated in the lung of MCS-exposed mice thereby counteracting an opposite effect triggered by MCS. In fact, the overall expression profiles in MCS-exposed mice treated with metformin tended to move away from those observed in MCS-exposed mice, in the absence of metformin, and to approach the situation recorded in both sham-exposed mice and metformin-treated mice. The upregulated miRNAs target a variety of mechanisms involved in pulmonary carcinogenesis, such as regulation of the cell cycle, modulation of *Ras*, *Myc*, *Dicer*, *Tgf-β*, and NF*κ*B, angiogenesis, stem cell recruitment, and multidrug resistance.

Given under conditions simulating an intervention in current smokers, exemplified by treatment of smoking diabetic patients, metformin was quite effective in protecting the lung from MCS-induced DNA alterations and in particular from oxidative DNA damage and formation of bulky DNA adducts. We cannot speculate whether inhibition of MCS-induced DNA adducts may be ascribed to metabolic mechanisms, since we did not find any study evaluating modulation of phase I or II enzymes by metformin. Having detected a high correlation between DNA adduct levels and 8-oxo-dGuo levels (*r* = 0.768, *P* < 0.001), it is possible that a proportion of MCS-related bulky DNA adducts may be related to oxidative DNA damage.

On the other hand, metformin failed to prevent the systemic clastogenic damage induced by MCS and development of lung adenomas and malignant tumors, which were evaluated 5 months after analyzing molecular biomarkers. Thus, while this drug proved to be effective in inhibiting the formation of surface lung adenomas induced by NNK, administered either orally or parenterally 14, it did neither affect the adenomas developed in mice treated with urethane^15^ nor the neoplastic lesions induced following inhalation of the whole complex mixture by mice (this study). On the other hand, we found that metformin significantly reduces both incidence and multiplicity of lung microadenomas, which are adenomas larger than hyperplastic foci but are only detectable at the microscope and tend to regress spontaneously 49. This finding may explain the failure of metformin to prevent lung tumors while inhibiting promutagenic lesions, such as DNA adducts, and preneoplastic alterations, such as microadenomas, which do not necessarily contribute to the development of malignant pulmonary lesions.

Metformin also inhibited MCS-induced hyperplasias of the tubular epithelium in kidney, which were totally suppressed in male mice treated with this drug. Note that renal hyperplastic lesions are probably the result of chronic irritation and might not lead to the development of renal cancer. Interestingly, metformin has been shown to protect against tubular cell injury in diabetic nephropathy via antioxidant mechanisms 50.

In conclusion, the analysis of early molecular biomarkers provided evidence that oral metformin is able to protect the mouse lung from MCS-induced DNA damage and to modulate a variety of miRNAs involved in pulmonary carcinogenesis. Although this anti-diabetic drug failed to prevent MCS-induced lung tumors, it inhibited preneoplastic lesions in both lung and kidney.

## Supporting Information

**Table S1.** Body weights (g) of Swiss H mice, as related to exposure to MCS and treatment with metformin.

**Table S2.** Survival, body weights (g) and frequency of MN NCE (‰) in Swiss H mice as related to exposure to MCS and to treatment with metformin with the diet.
