# Supplementary material for: Modulation by metformin of molecular and histopathological alterations in the lung of cigarette smoke-exposed mice
Source: Cancer Med. 2014 Mar 28;3(3):719–30. doi: 10.1002/cam4.234 (PMC4101764; doi:10.1002/cam4.234)
Supplement: Supplementary file 1 — Table S1. Body weights (g) of Swiss H mice, as related to exposure to MCS and treatment with metformin. [file cam40003-0719-SD1.doc]

**Supplementary Table S 1.** Body weights (g) of Swiss H mice, as related to exposure to MCS and treatment with metformin

|  |  | **Time after weaning (weeks)** | | | | | | | | | | | | | | | | | | | | | | | | | | | | | | |
| --- | --- | --- | --- | --- | --- | --- | --- | --- | --- | --- | --- | --- | --- | --- | --- | --- | --- | --- | --- | --- | --- | --- | --- | --- | --- | --- | --- | --- | --- | --- | --- | --- |
| **Treatment** | **Gender** | **0** | | |  | **1** | | |  | **2** | | |  | **3** | | |  | **4** | | |  | | **5** | | | |  | | **6** | | | |
| Sham | M | 23.9 | ± | 0.75 |  | 29.1 | ± | 0.87 |  | 31.5 | ± | 0.59 |  | 34.4 | ± | 1.03 |  | 37.1 | ± | 0.99 | |  | | 38.9 | ± | 0.72 | |  | | 40.0 | ± | 0.74 |
|  | F | 18.7 | ± | 0.87 |  | 23.7 | ± | 0.70 |  | 24.6 | ± | 0.65 |  | 27.3 | ± | 1.16 |  | 28.0 | ± | 0.78 | |  | | 28.2 | ± | 0.64 | |  | | 29.2 | ± | 0.56 |
|  |  |  |  |  |  |  |  |  |  |  |  |  |  |  |  |  |  |  |  |  | |  | |  |  |  | |  | |  |  |  |
| Metformin | M | 25.0 | ± | 1.19 |  | 31.5 | ± | 0.64 |  | 32.6 | ± | 0.65 |  | 34.9 | ± | 0.83 |  | 37.2 | ± | 1.82 | |  | | 38.4 | ± | 0.53 | |  | | 39.6 | ± | 0.90 |
|  | F | 19.8 | ± | 0.73 |  | 24.2 | ± | 0.62 |  | 25.6 | ± | 0.60 |  | 26.8 | ± | 1.17 |  | 28.6 | ± | 1.04 | |  | | 28.4 | ± | 0.69 | |  | | 28.9 | ± | 0.70 |
|  |  |  |  |  |  |  |  |  |  |  |  |  |  |  |  |  |  |  |  |  | |  | |  |  |  | |  | |  |  |  |
| MCS | M | 22.3 | ± | 0.50 |  | 27.8 | ± | 0.65 |  | 29.6 | ± | 0.66 |  | 31.4 | ± | 0.61^a^ |  | 33.0 | ± | 0.52^b^ | |  | | 33.2 | ± | 0.70^c^ | |  | | 33.9 | ± | 0.62^c^ |
|  | F | 17.6 | ± | 0.90 |  | 22.9 | ± | 0.38 |  | 23.7 | ± | 0.76 |  | 25.3 | ± | 0.72 |  | 25.8 | ± | 0.57^a^ | |  | | 26.2 | ± | 0.52^a^ | |  | | 27.3 | ± | 0.58^a^ |
|  |  |  |  |  |  |  |  |  |  |  |  |  |  |  |  |  |  |  |  |  | |  | |  |  |  | |  | |  |  |  |
| MCS +  Metformin | M | 22.5 | ± | 0.67 |  | 27.8 | ± | 0.64 |  | 29.7 | ± | 1.02 |  | 30.6 | ± | 0.75^a^ |  | 32.7 | ± | 1.24^b^ | |  | | 33.9 | ± | 1.00^c^ | |  | | 35.8 | ± | 1.35^a^ |
|  | F | 18.4 | ± | 0.67 |  | 22.9 | ± | 0.57 |  | 23.8 | ± | 0.86 |  | 25.6 | ± | 0.53 |  | 26.3 | ± | 0.89 | |  | | 27.1 | ± | 0.56 | |  | | 29.1 | ± | 0.72 |
|  |  |  |  |  |  |  |  |  |  |  |  |  |  |  |  |  |  |  |  |  | |  | |  |  |  | |  | |  |  |  |

The results are means ± SE of the body weights measured at each time point within each group of 5 mice.

Statistical analysis: *^a^P* < 0.05, ^b^*P* < 0.01, and ^c^*P* < 0.001, as compared with sham-exposed mice of the same gender.
