# Supplementary material for: Modulation by metformin of molecular and histopathological alterations in the lung of cigarette smoke-exposed mice
Source: Cancer Med. 2014 Mar 28;3(3):719–30. doi: 10.1002/cam4.234 (PMC4101764; doi:10.1002/cam4.234)
Supplement: Supplementary file 2 — Table S2. Survival, body weights (g) and frequency of MN NCE (‰) in Swiss H mice as related to exposure to MCS and to treatment with metformin with the diet. [file cam40003-0719-SD2.doc]

**Supplementary Table S 2.** Survival, body weights (g) and frequency of MN NCE (‰) in Swiss H mice as related to exposure to MCS and to treatment with metformin with the diet

|  |  |  | **After 1 month** | | | |  | **After 4 months** | | | | | | |  | **After 7 months** | | | |
| --- | --- | --- | --- | --- | --- | --- | --- | --- | --- | --- | --- | --- | --- | --- | --- | --- | --- | --- | --- |
| **Treatment** | **Gender** |  | **No. of mice  (% survival)** | **Body weight  (mean ± SE)** | | |  | **No. of mice  (% survival)** | **Body weight (mean ± SE)** | | | **MN NCE (mean ± SE)** | | | | **No. of mice (% survival)** | **Body weight (mean ± SE)** | | |
|  |  |  |  |  |  |  |  |  |  |  |  |  |  |  | |  |  |  |  |
| Sham | M |  | 45 (100) | 22.7 | ± | 0.46 |  | 45 (100) | 38.2 | ± | 0.62 | 1.3 | ± | 0.10 | | 44 (97.8) | 39.0 | ± | 0.67 |
|  | F |  | 49 (100) | 17.5 | ± | 0.40 |  | 48 (98.0) | 33.9 | ± | 0.37 | 0.9 | ± | 0.08 | | 45 (91.8) | 34.8 | ± | 0.39 |
|  |  |  |  |  |  |  |  |  |  |  |  |  |  |  | |  |  |  |  |
| MCS | M |  | 55 (100) | 16.7 | ± | 0.44^b^ |  | 52 (94.5) | 33.1 | ± | 0.38b | 1.9 | ± | 0.14^a^ | | 44 (97.8) | 38.6 | ± | 0.46 |
|  | F |  | 54 (100) | 15.5 | ± | 0.41^b^ |  | 54 (100) | 28.1 | ± | 0.47b | 1.2 | ± | 0.06^a^ | | 48 (87.3) | 35.9 | ± | 0.69 |
|  |  |  |  |  |  |  |  |  |  |  |  |  |  |  | |  |  |  |  |
| MCS + | M |  | 37 (100) | 17.5 | ± | 0.52^b^ |  | 36 (97.3) | 34.0 | ± | 0.64b | 2.2 | ± | 0.15^b^ | | 35 (94.6) | 35.2 | ± | 0.74^a,c^ |
| Metformin | F |  | 37 (100) | 15.6 | ± | 0.54^a^ |  | 37 (100) | 27.8 | ± | 0.46b | 1.2 | ± | 0.08^a^ | | 31 (83.8) | 34.3 | ± | 0.86 |

Statistical analysis: ^a^*P* < 0.01 and ^b^*P* < 0.001, as compared with sham-exposed mice of the same gender at the same time point. ^c^*P* < 0.001, as compared with MCS-exposed mice of the same gender at the same time point, in the absence of metformin
